# Sex-specific eNOS activity and function in human endothelial cells

**DOI:** 10.1038/s41598-017-10139-x

**Published:** 2017-08-29

**Authors:** Maria Grazia Cattaneo, Claudia Vanetti, Ilaria Decimo, Marzia Di Chio, Giuseppe Martano, Giulia Garrone, Francesco Bifari, Lucia Maria Vicentini

**Affiliations:** 1 Department of Medical Biotechnology and Translational Medicine, Università degli Studi di Milano, 20129 Milano, Italy; 2 Department of Diagnostics and Public Health, Università di Verona, 37134 Verona, Italy; 3 Institute of Neuroscience, CNR, 20129 Milano, Italy; 4 Fondazione IRCCS, Istituto Nazionale dei Tumori, 20133 Milano, Italy; 5 Laboratory of Cell Metabolism and Regenerative Medicine, Department of Medical Biotechnology and Translational Medicine, Università degli Studi di Milano, 20129 Milano, Italy

## Abstract

Clinical and epidemiological data show that biological sex is one of the major determinants for the development and progression of cardiovascular disease (CVD). Impaired endothelial function, characterized by an imbalance in endothelial Nitric Oxide Synthase (eNOS) activity, precedes and accelerates the development of CVD. However, whether there is any sexual dimorphism in eNOS activity and function in endothelial cells (ECs) is still unknown. Here, by independently studying human male and female ECs, we found that female ECs expressed higher eNOS mRNA and protein levels both *in vitro* and *ex vivo*. The increased eNOS expression was associated to higher enzymatic activity and nitric oxide production. Pharmacological and genetic inhibition of eNOS affected migratory properties only in female ECs. *In vitro* angiogenesis experiments confirmed that sprouting mostly relied on eNOS-dependent migration in female ECs. At variance, capillary outgrowth from male ECs was independent of eNOS activity but required cell proliferation. In this study, we found sex-specific differences in the EC expression, activity, and function of eNOS. This intrinsic sexual dimorphism of ECs should be further evaluated to achieve more effective and precise strategies for the prevention and therapy of diseases associated to an impaired endothelial function such as CVD and pathological angiogenesis.

## Introduction

There are substantial differences between men and women in cardiovascular disease (CVD) epidemiology, patho-physiology, clinical manifestations, effects of therapies, and outcomes^1–3^. However, the biological mechanisms responsible for these sex differences are not yet fully understood. An impairment in endothelial function is associated to all the common cardiovascular risk factors and is a hallmark of CVD development and progression^4, 5^. Endothelial dysfunction is triggered by a reduced endothelial Nitric Oxide Synthase (eNOS) activity with a consequent decrease in nitric oxide (NO) availability^6, 7^. Additional mechanisms of vascular NO formation *via* other NOS isoforms, such as the neuronal and inducible NOSs, may exist in blood vessels, and more generally in the CV system^6^. However, eNOS represents the main source of NO in endothelial cells (ECs) where it plays important roles in the control of several endothelial functions such as barrier integrity, vessel dilation, leukocyte adhesion, platelet aggregation, and angiogenesis^7, 8^. In ECs, eNOS is constitutively active under basal conditions and it can be further activated in response to shear stress, circulating hormones, and various autacoids^7^. Since females are more protected than males from CVD events until midlife, estrogens have long been assumed to mediate most of the observed sex-related differences^1–3, 9, 10^. Long term exposure to estrogens up regulates eNOS expression in ECs^11, 12^ whereas the activation of the enzyme and NO formation occur *via* rapid signaling pathways^13, 14^. Both the mechanisms participate in the improvement of EC function. As a result, flow mediated dilation (FMD), an indirect measure of NO-dependent endothelial function, is higher in women than in men until the early 50s^15^, and estrogen treatment improves FMD responses in recently postmenopausal women^16, 17^.

Recently, it has been demonstrated that more than 6.500 protein-coding genes are differentially expressed in men and women^18^. Regarding ECs, our and other groups have observed an intrinsic sexual dimorphism in some of their properties^19, 20^. Male and female ECs show sex-specific transcriptional profiles, with a higher expression of immune-related genes and a stronger response to shear stress in female compared to male ECs^20^. Knowledge of sex-specific expression patterns and of sexual dimorphisms in ECs may contribute to better understand the well-known physio-pathological differences in male and female endothelial function. In this study, we investigated eNOS expression both *in vitro* and *ex vivo* in human male and female ECs. Moreover, we studied the biological meaning of the observed sex-dimorphic eNOS expression by evaluating its involvement in relevant EC functions such as proliferation, migration, and *in vitro* angiogenesis.

## Results

### ENOS expression and activation are higher in female ECs

**Figure 1:**
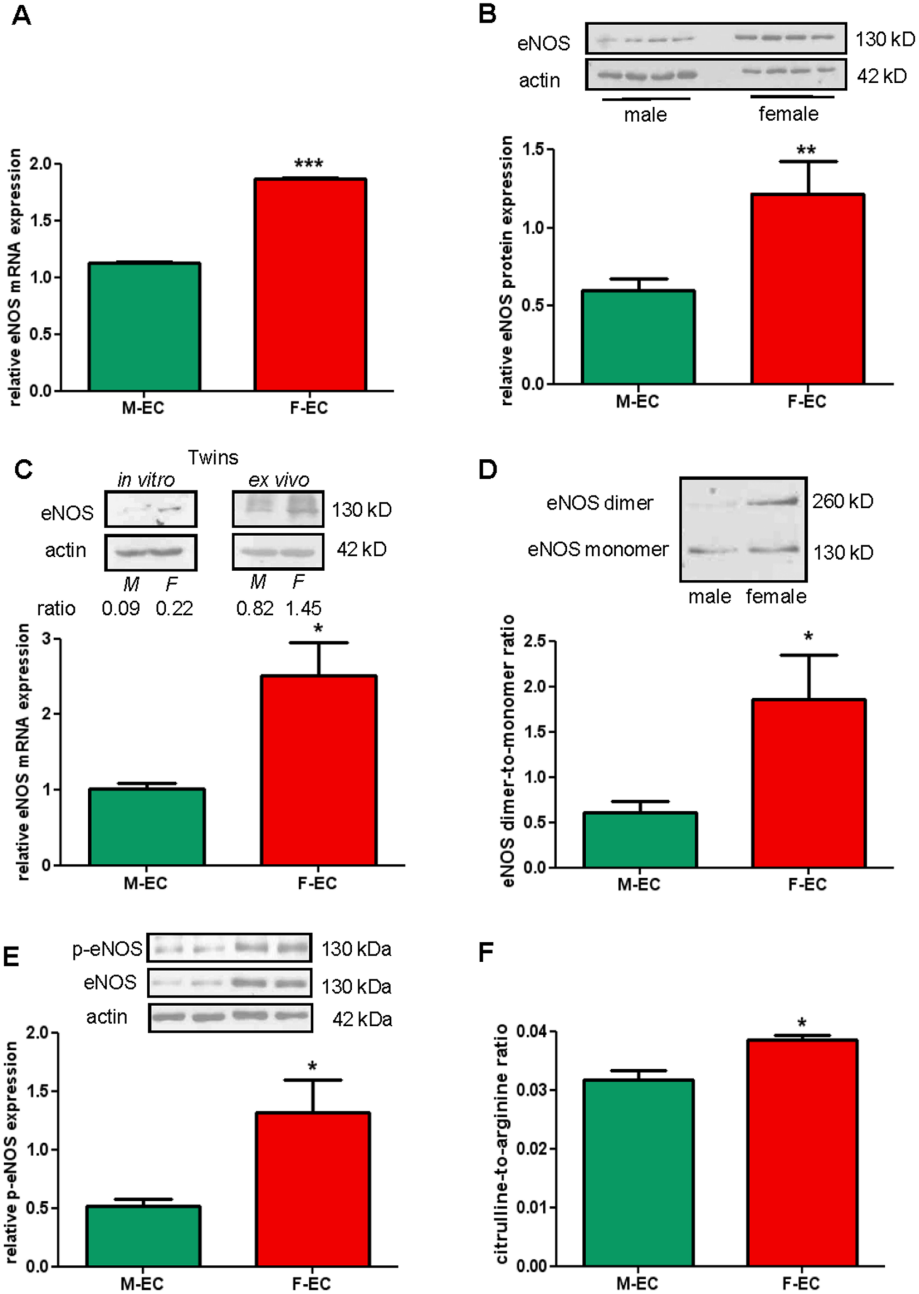
eNOS expression and activation are higher in F- than in M-ECs. (**A**) eNOS RNA was measured by RT-qPCR and normalized to the housekeeping gene 18 S. ***p < 0.001, n = 3. (**B**) Total eNOS protein was evaluated by immunoblotting on male and female lysates. β-actin was used as a loading control. A representative blot (showing 4 independent male and female lysates, 10 µg/lane) and the densitometric analysis of eNOS protein expression normalized to β-actin are shown. **p < 0.01; n = 14. (**C**) Upper panel, eNOS protein evaluated on lysates from twin male and female ECs at P1 passage (*in vitro*, left panel) or collected immediately after the flushing from cords (*ex vivo*, right panel). Representative blots out of two are shown. Lower panel, eNOS RNA measured by RT-qPCR on total RNA extracted from twin M- and F-ECs at P1 *in vitro* passage. Data are normalized to the housekeeping gene ACTB. *p < 0.05; n = 3. (**D**) eNOS dimers and monomers were separated by LT-PAGE as described in the Methods section. A representative blot and the densitometric analysis of the dimers-to-monomers ratio are shown. *p < 0.05; n = 6. (**E**) A representative blot (showing 2 independent male and female lysates, 20 µg/lane) and the densitometric analysis of phospho-eNOS^Ser1177^ (p-eNOS) protein expression normalized to β-actin are shown. *p < 0.05; n = 5. (**F**) LC-MS analysis was performed on M- and F-EC samples prepared and analyzed as described in the Methods section. Data are expressed as the citrulline-to-arginine ratio. *p < 0.05; n = 3.M- and F-ECs are green and red, respectively.

All cardiovascular risk factors are associated with a loss in endothelial functions that is triggered by a decrease in NO production as consequence of a reduced eNOS activity^6, 7^. For that reason, we investigated eNOS availability in male and female human umbilical vein endothelial cells (HUVECs, abbreviated as M-ECs and F-ECs, respectively). We started our experiments by analyzing eNOS expression in *in vitro* cultured ECs. We found that both eNOS gene and protein expression were about 2-fold higher in F- than in M-ECs (Fig. 1A and B). Notably, the relative higher amount of eNOS was detected in F-ECs also when M- and F-ECs were derived from dizygotic male and female twin pairs (Fig. 1C, upper left panel for protein, and lower panel for mRNA), thus suggesting that sex-related differences in eNOS levels did not depend on the exposure to potentially diverse maternal environment. To exclude that the increase in eNOS expression was secondary to *in vitro* culturing, we collected and analyzed ECs from twin umbilical cords *ex vivo*. Consistent with *in vitro* culture data, an increase in female eNOS protein levels was detected in dizygotic male and female twin ECs immediately lysed after the flushing from cords, *i.e*. without any *in vitro* passage (Fig. 1C, upper right panel).

To explore the functional status of eNOS, we first investigated the dimer-to-monomer ratio in M- and F-ECs. Monomeric (130 kDa) and dimeric (260 kDa) forms of eNOS have been described in various tissues and cells, and the formation of dimers is critically required for the enzyme to functionally produce NO^7, 21^. We found a significant increase in the dimer-to-monomer ratio in F- compared to M-ECs (Fig. 1D), suggestive of a correlation between the expression of eNOS and the amount of functional enzyme in F-ECs. Since a decrease in the dimer-to-monomer ratio may reflect eNOS uncoupling and production of reactive oxygen species (ROS)^7, 21^, we measured baseline ROS content in M- and F-ECs. However, we did not find any difference in ROS cell-associated levels between M- and F-ECs (Fig. S1), thus excluding that the lower dimer-to-monomer ratio in M-ECs may be due to a different oxidative behavior in comparison to F-ECs.

The phosphorylation of critical amino acids, such as Serine 1177 (Ser1177), contributes to the full activation of eNOS^7, 21^. Therefore, we analyzed baseline levels of phosphorylated eNOS (p-eNOS^Ser1177^) by using phospho-specific antibodies in immunoblot analysis. Remarkably, the amount of p-eNOS was about 2-fold higher in F- in comparison to M-ECs (Fig. 1E), further confirming a straight correlation between the level of expression and the activation of the enzyme in F-ECs.

We further assessed whether the increase in eNOS activity resulted in higher NO production. The formation of NO occurs through two oxidation steps catalyzed by eNOS. Initially, N-hydroxy-L-arginine is formed from L-arginine, and this intermediate is next converted to NO with the formation of L-citrulline as the byproduct^7, 21^. Therefore, we quantified by liquid chromatography-mass spectrometric (LC-MS) analysis the citrulline-to-arginine ratio in M- and F-ECs. Importantly, the higher expression and activation of female eNOS were accompanied by a significant increase in the citrulline-to-arginine ratio (Fig. 1F), indicating higher NO production in F-ECs.

Finally, to exclude a role for other NOS isoforms in NO formation, we measured both inducible and neuronal NOS protein levels (iNOS and nNOS, respectively) in M- and F-EC lysates (Fig. S2). We could neither find iNOS nor nNOS expression in ECs from both sexes, thus excluding their involvement in the observed phenotype.

### EC proliferation is eNOS-independent in both sexes

**Figure 2:**
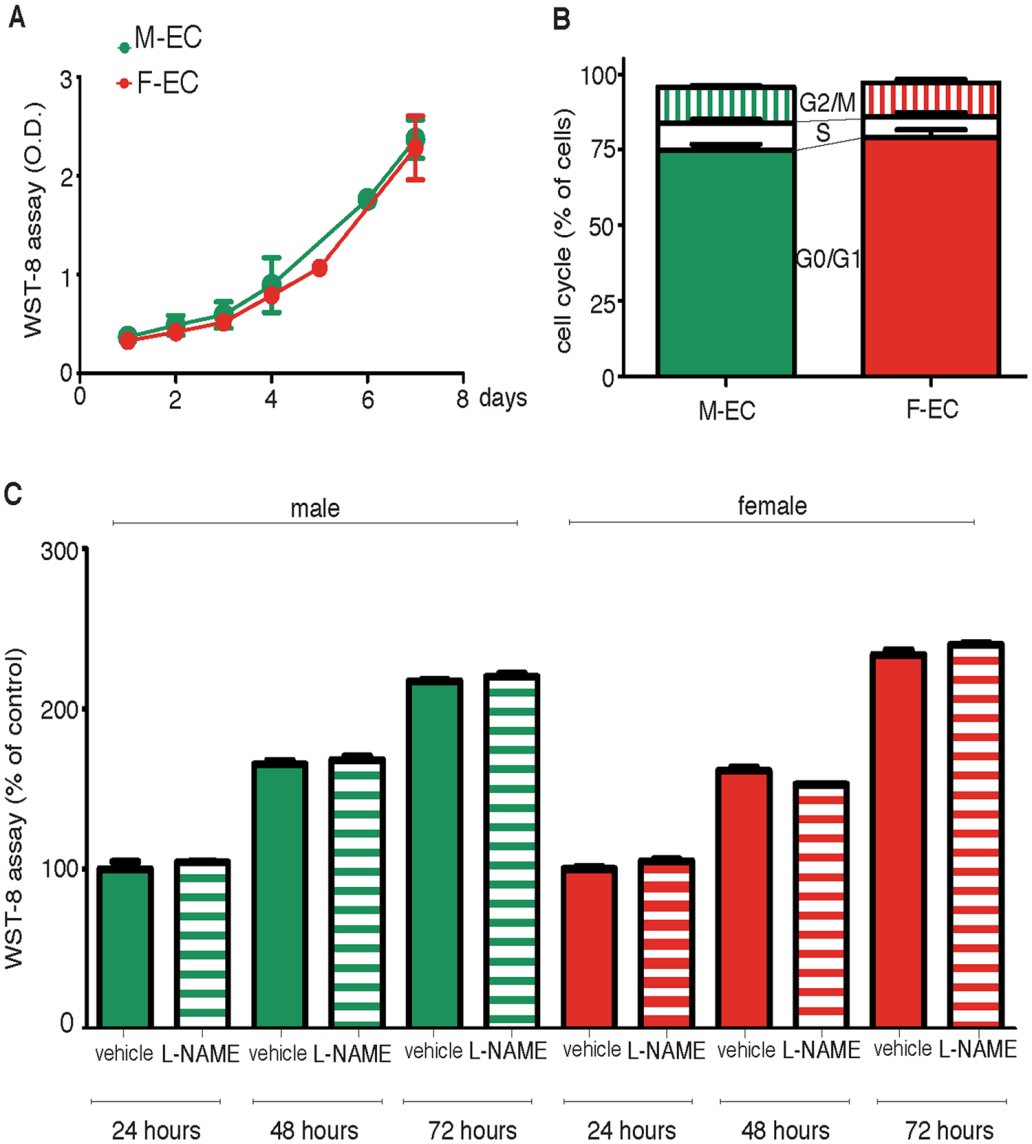
EC proliferation is eNOS-independent in both sexes. (**A**) M- and F-ECs were cultured for the indicated times, and WST-8 absorbance was measured after 4 h of incubation. n = 3. (**B**) Cell cycle distribution was analyzed by FACS 48 h after plating. The percent of cells in each phase of the cycle is shown (solid, G0/G1; open, S; vertical, G2/M). n = 3–4 for M- and F-ECs, respectively. (**C**) M- and F-ECs were cultured in the absence (solid bars) or in the presence of L-NAME (100 µM, horizontal bars), and WST-8 was added for the last 4 h of incubation. Data are expressed as the percent of absorbance measured in the corresponding vehicle-treated cells. n = 3. M- and F-ECs are green and red, respectively.

To find a biological significance for the increased female expression of eNOS, we compared proliferative properties of M- and F-ECs, and their dependence on eNOS activity by using L-NAME, a well-known functional NOS inhibitor. We did not observe any difference in growth rates of M- and F-ECs (Fig. 2A). Both M- and F-ECs showed comparable doubling times (47.5 ± 5.4 and 47.6 ± 6.0 h for M- and F-ECs, respectively). Moreover, cell cycle distribution analysis showed a similar percent of cells in G1/S or G2/M phases (Fig. 2B). The addition of L-NAME (100 µM) did not modify cell growth at any time tested both in M- and in F-ECs (Fig. 2C).

### Female ECs show higher eNOS-dependent migratory properties than male ECs

**Figure 3:**
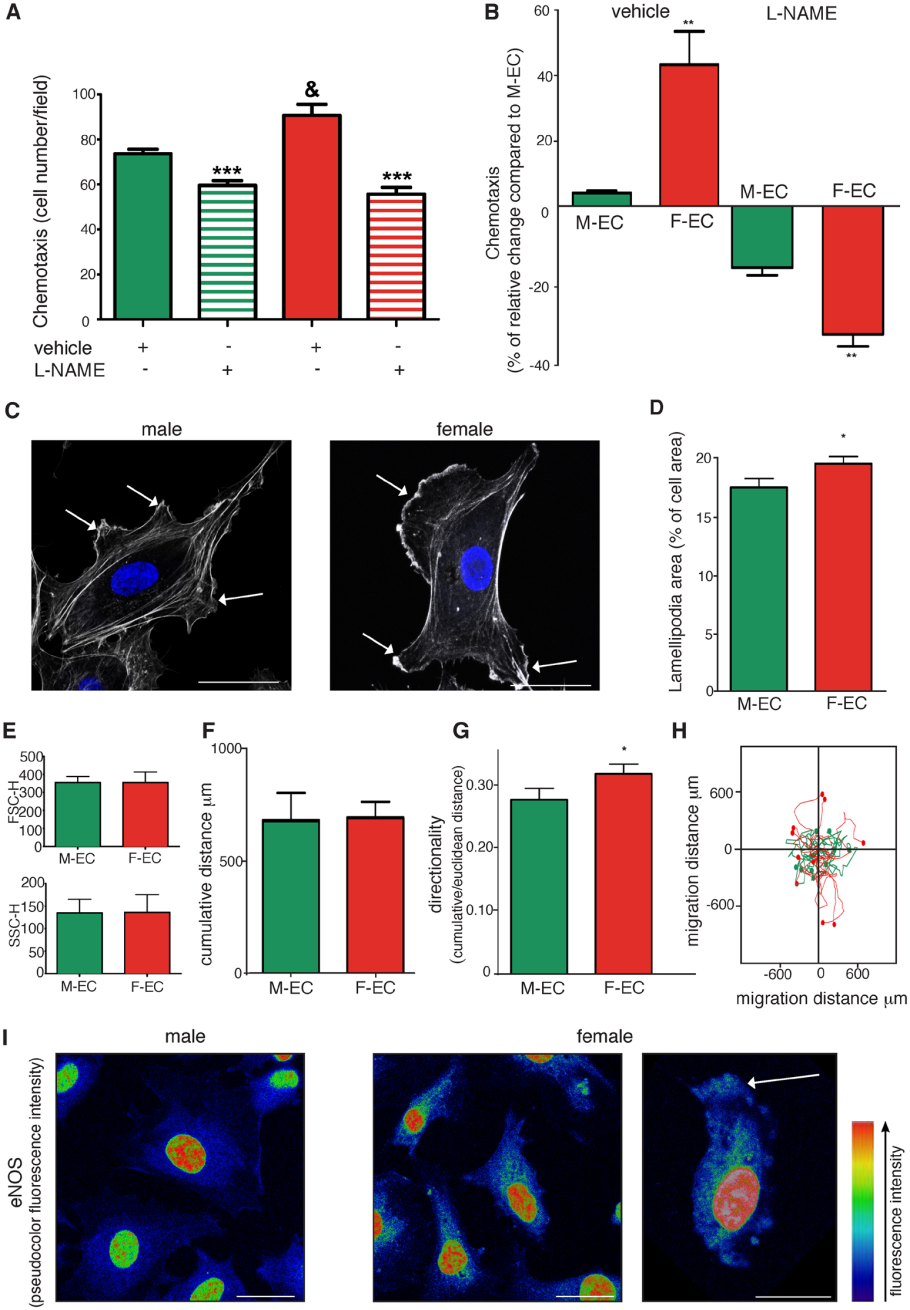
F-ECs show higher eNOS-dependent migratory properties than M-ECs: correlation with lamellipodia and eNOS localization. (**A**) Chemotaxis toward 10% FBS was performed in the absence (solid bars) or in the presence of L-NAME (100 µM, horizontal bars). Results are expressed as the number of migrating cells, and are the mean ± s.e.m of 25 counts from a representative experiment, repeated 3 times with similar results. ***p < 0.001 *vs *vehicle, ^&^p < 0.05 *vs* M-ECs. (**B**) Results are expressed as the percent of change in the number of migrating F-ECs over M-ECs in the presence of vehicle (**p < 0.01, n = 4) or L-NAME (**p < 0.01, n = 3). (**C** and **D**) Representative images of FITC-labeled phalloidin-stained male (left panel) and female (right panel) showing lamellipodia (white arrows), and quantification of the area occupied by lamellipodia expressed as the percent of the total cell area. *p < 0.05, n = 3. Scale bar, 20 µm. (**E**) Forward scatter height (FSC-H) and side scatter height (SSC-H) of M- and F-ECs. n = 8 for M-ECs, and n = 9–10 for F-ECs. **(F, G** and **H)** Analysis of spontaneous cell-motility tracks by time-lapse imaging in M- and F-ECs, showing quantification of the cumulative distance, directionality, and migration distance. *p < 0.05, n = 108–178 for M- and F-ECs across 6 different experiments. (**I**) Representative pseudocolor lookup table (LUT) high-magnification confocal images of male and female ECs stained with anti eNOS highlighting the enzyme localization at the leading edge of migrating female ECs (white arrow). Scale bar, 20 µm.

Having excluded the involvement of eNOS in proliferation, we analyzed its contribution to migration, another crucial property characterizing EC phenotype^22^. When EC motility was evaluated in a chemotaxis assay using FBS as attractant, F-ECs showed a greater migratory capability in comparison to M-ECs (Fig. 3A and B). The treatment with L-NAME (100 µM) completely abolished the higher F-EC migratory response bringing it to the same level of M-ECs (Fig. 3A). Although partially present also in M-ECs, the L-NAME-induced inhibition resulted significantly higher in F-ECs (−32.6 ± 3.0% *vs* −15.7 ± 1.9% for F- and M-ECs, respectively) (Fig. 3B).

Since the establishment and maintenance of directional motility in chemotactic migration require lamellipodia formation at the leading edge of migrating cells^23–25^, we compared lamellipodia extension between M- and F-ECs. Measurement of lamellipodial area revealed that F-ECs showed a significantly higher percent of the total cell area occupied by lamellipodia than M-ECs (Fig. 3C and D). No obvious differences were observed between cellular size (FSC-H) and complexity (SSC-H) (Fig. 3E), thus excluding broad differences between M- and F-EC morphology.

Finally, we tracked random cell-motility of individual ECs by video imaging. This analysis revealed that, although M- and F-ECs showed a comparable cumulative distance (Fig. 3F), F-ECs displayed a greater directional persistence of movement than M-ECs (Fig. 3G and H).

NO is a short-lived molecule and therefore the cellular compartmentalization of eNOS is crucial to determine its distribution and biological effects^7, 8^. For this reason, we assessed the cellular localization of eNOS in M- and F-ECs. In agreement with functional and morphological results, we observed a more pronounced accumulation of the enzyme at the leading edge of polarized F-ECs (Fig. 3I). Collectively, all these results suggest that F-ECs possess higher migratory capabilities in comparison to M-ECs. These properties show a morphological relationship with the presence of lamellipodia and a functional correlation with the activity and localization of eNOS.

### 2-D wound healing assay reveals eNOS-dependent migration in female ECs

**Figure 4:**
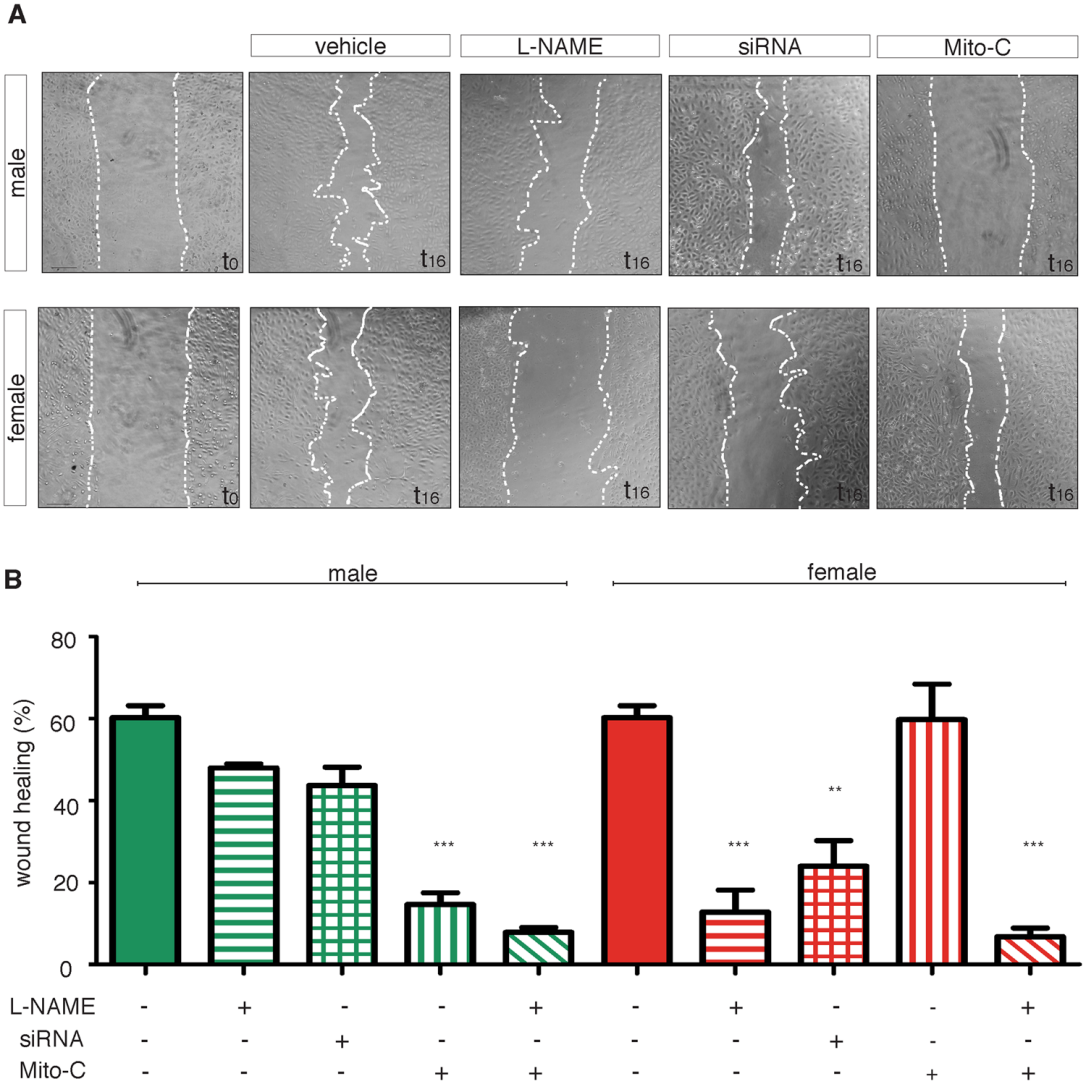
2-D wound healing assay reveals eNOS-dependent migration in female ECs. (**A**) Representative photographs of male and female ECs taken at the time of wounding (t 0) and after a 16- h incubation in 20% FBS (vehicle) or in 20% FBS supplemented with L-NAME (100 µM) or mytomicin (MitoC, 500 ng/ml). Images of male and female ECs transfected with eNOS siRNA 48 h before wounding and then incubated for 16 h in 20% FBS are also shown. Scale bar, 100 µm. **(B)** The percentage of wound closure at 16 h is shown. Vehicle, solid bars; L-NAME, horizontal bars; siRNA, squared bars; MitoC, vertical bars; L-NAME + MitoC, diagonal bars. **p < 0.01; ***p < 0.001 *vs* the corresponding vehicle, n = 3–4 for M- and F-ECs, respectively.

Migration was also studied by using a 2-D wound healing test. In this assay, we did not observe any difference in the percentage of wound closure – measured 16 h after the scratch - between control M- and F-ECs (60.2 ± 3.0% and 56.7 ± 4.1%, respectively) (Fig. 4A and B). To assess the role of eNOS-mediated migration in this assay, we added L-NAME (100 µM) to block the enzyme. We found that L-NAME was much more effective in inhibiting the percentage of wound closure in F- than in M-ECs. Indeed, the ability to close the wound was inhibited by 80% in L-NAME-treated F-ECs whereas it was reduced in a non significant manner in M-ECs (Fig. 4A and B). To test if the effect of L-NAME was attributable to its ability of specifically inhibiting eNOS activity, the expression of the enzyme was silenced by using siRNA, and 48 h after transfection a scratch assay was performed. The knock-down of eNOS reduced the percentage of wound closure by about 50% in F-ECs whereas it did not significantly affect the capacity of M-ECs to close the wound (Fig. 4A and B). The ability of siRNA to efficiently silence eNOS expression in both M- and F-ECs is shown in Fig. S3.

Wound healing is the result of a complex process, which relies on both cell migration and proliferation. To extrapolate the contribution of cell growth to the closure of *in vitro* wounds, we performed the scratch assay on M- and F-ECs mitotically arrested by the addition of mitomycin (Mito-C, 500 ng/ml). Surprisingly, M-ECs became unable to close the wound when proliferation was blocked by mitomycin (Fig. 4A and B). On the other hand, inhibition of proliferation did not affect the ability of F-ECs to close the gap (Fig. 4A and B). Indeed, the percentage of wound closure was reduced by about 80% in mitomycin-treated M-ECs, while it remained unchanged in growth-arrested F-ECs (Fig. 4B). These results suggest that the ability to migrate and to close the wound significantly differs between M- and F-ECs. The activity of eNOS is a crucial requirement for F-EC migration and wound closure. At variance, M-EC wound closure is dependent on proliferation and is not significantly affected by eNOS inhibition.

### eNOS is essential for *in vitro* angiogenesis of female ECs

**Figure 5:**
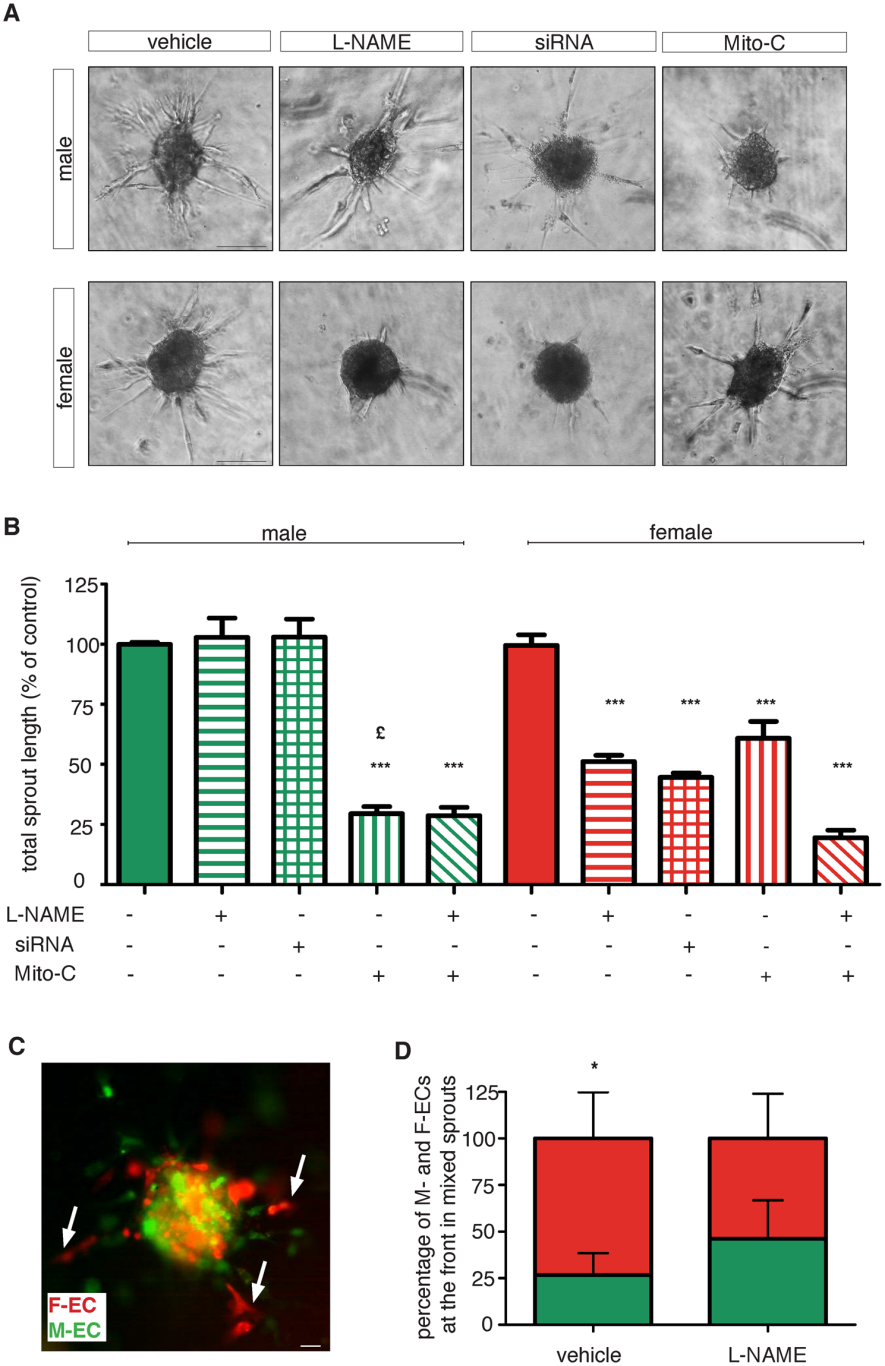
eNOS is essential for *in vitro* angiogenesis in female ECs. (**A**) Representative images of spheroids from male or female ECs embedded in collagen gels in the absence (vehicle) or in the presence of L-NAME (1 mM) or MitoC (500 ng/ml). Also eNOS siRNA-transfected spheroids are shown. Photographs were taken 18 h later. Scale bar, 100 µm. (**B**) Quantification of *in vitro* angiogenesis. Results are expressed as the percent of the total sprout length measured in vehicle–treated cells, set at 100%. Vehicle, solid bars; L-NAME, horizontal bars; siRNA, squared bars; MitoC, vertical bars; L-NAME + MitoC, diagonal bars. ***p < 0.001 *vs* the corresponding vehicle, ^£^p < 0.05 *vs* MitoC-treated F-ECs, n = 4–7 for M- and F-ECs, respectively. (**C**) Representative fluorescence photograph of a mosaic spheroid containing a 1:1 mixture of GFP-M-ECs and Cherry-F-ECs embedded in collagen gels. Photographs were taken 18 h later. Arrows indicate F-ECs located as tip cells. Scale bar, 100 µm. (**D**) Quantification of the percentage of tip cell contribution in sprouts containing both GFP-M-ECs and Cherry-F-ECs emerging from vehicle- or L-NAME-treated spheroids. *p < 0.05, n = 17.

Angiogenesis is the result of a coordinated process where the growth of new blood vessels relies not only on the migration of specialized cells at the forefront of vascular sprouts – the tip cells – but also on trailing cells that undergo proliferation to elongate the branch – the stalk cells^26^. Therefore, we set up a 3-D spheroid assay that recapitulates *in vitro* the angiogenic process^27^. In this assay, spheroids from M- or F-ECs undergo sprouting in a 3-D collagen matrix as the result of the migratory and proliferative contributions. Both spheroids from M- and F-ECs efficiently spread after 18 h of incubation (Fig. 5A), and no differences were observed in their overall total sprout length (1990 ± 315 and 1962 ± 275 µm for M- and F-ECs, respectively). To dissect eNOS-dependent migration and proliferation activities in EC sprouting, we either pharmacologically and genetically inhibited eNOS or mitotically arrested the cell cycle by the addition of mitomycin. When we added L-NAME (1 mM), we observed inhibition of sprouting only in F-EC spheroids (Fig. 5A and B). Quantification of the total sprout length showed a reduction of about 50% in F-ECs whereas M-ECs were totally unaffected (Fig. 5A and B). Comparable results were obtained when M- or F-ECs were silenced for eNOS gene expression (Fig. 5A and B). Addition of mitomycin (Mito-C, 500 ng/ml) impaired sprouting in both M- and F-ECs (Fig. 5A and B). However, the inhibitory effect on sprouting was significantly higher in spheroids from growth-arrested M-ECs (about 70%) in comparison to spheroids from mitomycin-treated F-ECs (about 40%) (Fig. 5B). When we blocked both eNOS activity and proliferation, we observed a comparable inhibition of sprouting in M- and F-ECs (Fig. 5B). All together, these results suggest that F-ECs are crucially dependent on eNOS activity not only for their migratory phenotype but also for *in vitro* angiogenesis. At variance, M-ECs appeared to rely more on proliferation for both the events.

Our results obtained by studying migration and *in vitro* angiogenesis suggest that F-ECs possess a better migratory phenotype when compared to M-ECs. We therefore challenged the ability of M- and F-ECs to migrate during *in vitro* sprouting assay by performing experiments with mosaic spheroids prepared by mixing in a 1:1 ratio GFP-carrying M-ECs and Cherry Red-expressing F-ECs (Fig. 5C). Quantification of the number of sprouts emerging from mixed spheroids showed that the majority of sprouts segregated only M- or F-ECs. However, in sprouts where ECs of both sexes were present, Cherry-F-ECs migrated more distally in about 73% of the sprouts (Fig. 5D). Notably, when mosaic spheroids were incubated in the presence of L-NAME (1 mM), the tip cell activity showed by F-ECs was significantly reduced to about 50% by the treatment (Fig. 5D). Again, our results confirm the hypothesis of a higher migratory phenotype shown by F-ECs and strictly controlled by eNOS activity.

## Discussion

In this study, we show an intrinsically sexual dimorphism of the expression and activity of eNOS in human ECs. We have found that F-ECs possess a higher eNOS expression than M-ECs when analyzed *ex vivo*, *i.e*. before tissue culture, and that this difference is maintained in cell culture. Interestingly, the higher eNOS expression in F-ECs was confirmed when fraternal male and female twin pairs were analysed, thereby suggesting that the increased female eNOS expression is independent of the maternal environment.

Since sex hormones, especially estrogens, are well-known positive regulators of eNOS expression in ECs^11, 12^, it is possible to hypothesize that some differences in their fetal and/or maternal levels might be responsible for the increased eNOS expression in F-ECs. However, it has been shown that fetal cord blood contains steroids, including 17β-estradiol, but their levels are comparable between male and female fetuses^28, 29^. Moreover, studies in twin pregnancies confirm that maternal serum steroid levels are unrelated to the fetal sex^30^. Therefore, the increased inborn female eNOS expression seems to be independent of estrogen exposure and concentrations. Whether other molecules of maternal and/or fetal origin might be responsible for the observed sexual dimorphism of eNOS expression is still unknown. Nevertheless, our data indicate that such sex-specific eNOS expression is maintained *in vitro* through sequential passages, thus suggesting a genetic/epigenetic regulation of this feature, and supporting the hypothesis that HUVECs have a sex.

It has been recently demonstrated that more than 6.500 protein-coding genes are differentially expressed in men and women^18^. Sexually dimorphic properties mainly result from differential expression of genes present in both sexes^18^, and it has been proposed that sex-specific biological features are the resultant of evolutionary pressure^31^. Moreover, sex rather than cell type represents the main driver behind overall DNA methylation levels in pluripotent cell lines^32^. Interestingly, sex-differential expression of genes may be related to different disease susceptibility^18^. The higher expression of immune genes and the stronger capacity of inducing the differentiation of maternal regulatory T cells shown by female HUVECs compared to male HUVECs might be associated with the greater immune reactivity and predisposition for autoimmune responses observed in females in comparison to males^20, 33^. Our results suggest that eNOS, in addition to the well-known estrogen-dependent stimulation^1–3, 9, 10^, is constitutively expressed in an inborn sex-specific manner. The reduced incidence of CVD in premenopausal women relative to age-matched men has been largely attributed to the vascular protective effect of estrogens^1–3, 34, 35^, and their menopausal loss is accompanied by a progressive decline in endothelial function^9, 10^. How the estrogen-dependent component of the eNOS/NO production might interact *in vivo* with the innate, constitutive eNOS/NO component during lifetime, and whether this may contribute to the protection against CVD of premenopausal female population, is however still unknown. Similarly, whether and through which mechanisms the expression of eNOS is lifelong regulated in male and female ECs need to be investigated.

Importantly, the increased eNOS expression observed in F-ECs corresponds to higher levels of activated enzyme. Furthermore, higher functional dimers and phosphorylated eNOS are associated to an increment in the citrulline-to-arginine ratio in F-ECs, thereby confirming a greater basal release of NO in F-ECs. A higher basal release of NO has been observed in aortic rings from endothelium of female rabbits compared to males^36^. A more robust endothelium-dependent vasodilation due to higher rates of NO release has also been described in premenopausal women compared to age-matched men^37^. Notably, our results demonstrate that, besides expression, also an increased eNOS activity and subsequent NO formation are constitutive properties of F-ECs.

eNOS is involved in several relevant EC functions including migration and angiogenesis^7, 8, 22, 38^. Pharmacological acute inhibition of eNOS activity with L-NAME impairs EC chemotaxis^22, 39–41^, and systemic administration of the drug blocks *in vivo* angiogenesis^39, 40^. Also the genetic loss of eNOS impairs *in vivo* Vascular Endothelial Growth Factor (VEGF)- or ischemia-induced angiogenesis^42, 43^. In accordance with the permissive role of eNOS on cell movement, our data show that the higher eNOS expression and activity were accompanied by an increased motility in F-ECs in comparison to M-ECs. Although eNOS appeared to be required for both M- and F-EC chemotaxis, pharmacological inhibition of the enzyme revealed a much higher dependence on eNOS for F-EC migration. Since NO is a short-lived molecule, the site of its production determines its biological effects^7, 8^. In mixed-sex EC cultures, the active form of eNOS specifically localizes to lamellipodia at the leading edge of approximately half of the migrating cells^44, 45^, and polarization of eNOS and NO signalling (*via* generation of cGMP by NO-activated soluble guanylyl cyclase) have been demonstrated to define the direction of migration^45^. Consistently, our results show that F-ECs *i)* form lamellipodial projections over large areas; *ii)* have a higher eNOS concentration at the leading edge of polarized cells; *iii)* show a better directional motility than M-ECs.

The difference in eNOS dependence observed between M- and F-EC migration has been highlighted in the wound healing assay, where the spatial spreading of cells involves both motility and proliferation. Only F-ECs strictly required eNOS expression and activity for the closure of the wound. At variance, M-ECs spread in the wound region also when eNOS was inhibited or silenced but failed to close the gap when ECs were mitotically arrested.

A fine-tuned balance between cell growth and migration also underlies angiogenesis. Lamellipodia formation and motility of tip cells at the forefront of vascular sprouts are critical for vessel outgrowth and branching whereas elongation of the branch are sustained by proliferation of stalk cells^26^. Although we did not observe any sexual dimorphism in the overall sprouting ability of M- and F-ECs, we confirmed relevant sex-specific differences in eNOS dependence also in the *in vitro* angiogenic process. Again, only F-EC sprouting relied on eNOS expression and activity whereas capillary outgrowth was unaffected by eNOS inhibition in M-ECs. *Vice versa*, M-EC sprouting was significantly inhibited when proliferation was blocked by mitomycin. Overall, our results demonstrate that M-ECs utilize eNOS-independent proliferation as the main mechanism for wound healing and sprouting. On the contrary, F-ECs were highly dependent on eNOS expression and activity for both the *phenomena*.

A major difference between M- and F-ECs is represented not only by the level of expression of eNOS but also by its localization. Lamellipodial eNOS is strictly required for EC motility and sprouting^44, 45^ and, in our experiments, mainly migrating F-ECs showed eNOS localization at the leading edge. Indeed, the pool of eNOS achievable for activation resides within the plasma membrane, and is enriched in specialized domains where several proteins can interact with eNOS to regulate its activity. Among these proteins, calcium-activated calmodulin and heat shock protein 90 (hps90) activate eNOS whereas caveolin-1 acts as a scaffold by inhibiting eNOS. An altered caveolin-1 membrane expression has been associated to a reduced eNOS level and activity in the plasma membrane^46^. More recently, changes in the lipid component of EC membranes have been shown to affect the sub cellular distribution of eNOS and its activation by different *stimuli* ^47^. It is therefore possible to speculate that, besides eNOS, some differences in the expression and/or function of eNOS-interacting proteins, as well as in the lipid component of cell membranes, might also exist between M- and F-ECs leading to a different segregation of the enzyme among diverse cellular compartments. This hypothesis is currently under investigation in our laboratory.

In conclusion, our study shows for the first time a sexual dimorphism in the expression, activity, and function of eNOS in M- and F-ECs. Sex-differential expression of genes has been recently related to different disease susceptibility^18^. Nevertheless, biological sex is still under investigated in preclinical and *in vitro* studies, even when studies on sex-dependent pathologies are performed^48–50^. From this perspective, our results, providing new insights in sex-specific EC properties, may implement sex-related differences into health care strategies. A better understanding of EC dimorphisms and of their mechanisms may further suggest new targets for the design of more precise preventive and therapeutic strategies for diseases associated to an impaired endothelial function such as CVD and pathological angiogenesis.

## Methods

### Ethical approval

The procedure was carried out in accordance to local university guidelines and with the principles outlined in the Declaration of Helsinki. All experimental protocols were approved by the Ethics Board at the University of Milano (study 106/2011). Collection of cords was conducted by the clinicians of the Ospedale Macedonio Melloni, 20129 Milano, Italy, and anonymized samples were processed at the Dept of Medical Biotechnology and Translational Medicine, University of Milan, Milano, Italy. All pregnant women gave their written informed consent to study participation.

### Cell cultures

HUVECs were freshly isolated from umbilical cords as described^51^, and used at passages 1–3. Cells were routinely grown in 199 medium supplemented with 20% fetal bovine serum (FBS), 25 µg/ml endothelial cell growth supplement (ECGS), and 50 μg/ml heparin on 0.1% gelatine-coated surfaces. When HUVECs were immediately collected for *ex vivo* experiments, contaminating red blood cells were removed by red blood cell lysis buffer. Except twins, we used HUVECs pooled from two or more donors to minimize the variability associated with cells derived from a single male or female newborn donor.

### Total RNA extraction for reverse transcription and quantitative real time PCR (RT-qPCR)

Total RNA was extracted using the RNeasy® Mini Kit and accompanying QIAshredder™ (Qiagen). To avoid DNA contamination of samples, a 15 min on-column incubation was carried out with DNase I (Qiagen). Reverse transcription was performed using the SuperScript™ III First-Strand Synthesis System (Thermo Fisher Scientific). For the quantitative analysis of gene expression we used the ABI Prism® 7000 Sequence Detection System or the StepOnePlus® Real-Time PCR System (Thermo Fisher Scientific). Target sequences were amplified from 50 ng of cDNA using the TaqMan® Primer and Probe assays for human eNOS (NOS3, Hs00167166_m1) and for the endogenous control 18 S (Hs99999901_s1) (Thermo Fisher Scientific), or the PrimeTime® qPCR assays for eNOS (NOS3, Hs.PT.58523162) and actin (ACTB, Hs.PT.39a.22214847) (IDT). For calculation of results, the 2^−ΔΔCt^ method was used.

### Immunoblotting

Western blots for total and phospho-eNOS were carried out on total cell lysates prepared in Laemmli sample buffer containing 1 mM sodium orthovanadate. Equal amounts of proteins were separated by standard 8% SDS-PAGE. For the detection of eNOS dimers and monomers, HUVECs were lysed as previously described^52^, and the same amount of proteins (10 µg/lane) was subjected to low temperature 6% SDS-PAGE (LT-PAGE, performed by keeping all gels and buffers at 4 °C during the whole procedure) under reducing conditions (2.5% 2-mercaptoethanol)^52^. Proteins were then transferred onto nitrocellulose membranes following standard procedures. Membranes were blocked for 1 h with 5% non fat dried milk in Tris-buffered saline containing 0.05% Tween-20 (TBS-T), and probed overnight at 4 °C with the following primary antibodies: anti-total eNOS, 1:2000 in 5% milk in TBS-T; anti-phospho eNOS (Ser1177) 1:1000 in 5% bovine serum albumin (BSA) in TBS-T; anti-β-actin, 1:1000 in 5% milk in TBS-T. After incubation with the appropriate HPR-conjugated secondary antibody (1:10.000 in 5% milk in TBS-T), immunoreactive bands were visualized by chemiluminescence (LiteAblot Turbo, EuroClone). Densitometric analyses of immunoblots were performed using the National Institute of Health (NIH) Image J software package. Full length blots are shown in Fig. 1.

### Liquid chromatography-mass spectrometric (LC-MS) analysis

ECs cultured on 13-mm diameter glass coverslips were rapidly washed with a steam of water and immediately subjected to metabolic quenching as described in details in Martano *et al*.^53^, using ^u13^C-Glucose as internal standard. Dry samples were reconstituted in 100 µl of 90% acetonitrile, 0.05% acetic acid and 0.025% trifluoroacetic acid, vortex for 30 seconds, and centrifuged at 3.500 g at 4 °C for 10 minutes. After a 2-min sonication, samples were centrifuged again, and supernatants were injected for analysis. Chromatographic separation was performed with Transcendent LX System (Thermofisher Scientific) using a Luna-Hilic column (particle size 3 µm, id 2.00 × 100 mm, Phenomex). Acquisition was performed with a triple quadrupole mass spectrometer TSQ-Quantiva (Thermofisher Scientific) coupled with electro-spray ionization source. Isocratic separation was performed as described^54^ by using 3% mobile phase A with MilliQ, 0.5% acetic acid, 0.025% trifluoroacetic acid, and 97% mobile phase B with acetonitrile, 0.5% acetic acid, 0.025% trifluoroacetic acid. Injection was performed in full loop using 5 µl loop. MS parameter are: positive ion (V), 4.500; sheath gas, 45; aux gas, 15; sweep gas, 0; ion transfer tube temperature, 275 °C; vaporizer temperature, 150 °C. Ions for selected reaction monitoring (SRM) are L-arginine: 116.20, 70.10, 60.40; L-Citrulline: 159.05, 113.14, 70.10; ^u13^C-Glucose: 169.08, 150.06.

### Small interfering RNA (siRNA) transfection

To silence eNOS expression, HUVECs were transfected with validated Stealth^TM^ siRNA duplexes against human eNOS (GC content 48%, Thermo Fisher Scientific). A negative control duplex (Medium GC Duplex, 48% GC content, Thermo Fisher Scientific) was used as control. Both siRNAs were individually transfected at a 10 nM concentration using the PepMute transfection reagent according to the manufacturer’s instructions (SignaGen Laboratories). For scratch and sprouting assays, cells were transfected 48 h before wounding, and 24 h before plating to form spheroids, respectively. The knockdown of eNOS expression was analyzed by immunoblotting.

### Proliferation assay

HUVECs were seeded in 96-well plates in triplicate at a density of 2 × 10^3^ cells/well, and proliferation was measured by WST-8 assay according to the manufacturer’s instructions (Dojindo).

### Flow cytometry analyses

For cell cycle analysis, HUVECs were re-suspended at a density of 1 × 10^6^ cells/ml in PBS containing 0.1% Nonidet, RNAase A (100 µl, stock 100 µg/ml), and propidium iodide (800 µl, stock 50 µg/mL). After a 30-min incubation in the dark, cells were analyzed using a FACScalibur flow cytometer (BD Biosciences) operated by Cell Quest software. To study cell size and complexity, forward-scatter height (FSC-H) and side-scatter height (SSC-H) were acquired.

### Motility assays

Cell migration was detected by chemotaxis experiments in Boyden chamber as previously described^41^. Briefly, HUVECs were suspended at a density of 1 × 10^6^ cells/ml in 199 medium with 1% BSA and allowed to migrate toward 10% FBS as attractant. Polyvinylpyrrolidine (PVP)-free polycarbonate filters (8 μm pore size, Neuroprobe) were coated with type I collagen (10 µg/ml). After a 6-h incubation at 37 °C, the cells that had migrated to the lower side of the filter were stained with Diff-Quick stain, and 5 unit fields per filter were counted by a scorer blind to the experimental conditions using a Zeiss microscope.

For the wound healing assay, a scratch wound was applied on confluent HUVECs using a sterile pipette tip. HUVECs were then incubated in 199 medium with 20% FBS, and images were captured immediately after wounding (T_0h_) and 16 h later (T_16h_) at a 10x magnification with an Olympus U-CMAD3 phase contrast microscope equipped with an Olympus digital camera. When indicated, cells were mitotically arrested with mitomycin (500 ng/ml overnight). The percentage of wound closure (gap area at T_0_ *minus* gap area at T_16h_/gap area at T_0_ × 100) was measured with the NIH Image J software package.

Spontaneous motility was assessed on sparse HUVECs plated on glass bottom 6-well plates in 199 medium with 20% FBS. Images were acquired at intervals of 30 min overnight at 37 °C using a Evos FL Auto Cell Imaging System (objective ×10; Thermo Fisher Scientific). The motility of the cells was analyzed using the Tracking Tool PRO v2.0 Software (Gradientech AB) by tracking nucleus position over time and by superimposing migration origin at the zero-cross point^55^.

### Immunofluorescence

For lamellipodia analysis, HUVECs were fixed in 4% paraformaldehyde (PFA)/0.12 M sucrose, and permeabilized with 0.3% Triton X-100. Fluorescein isothiocyanate (FITC)-labelled phalloidin was used to detect filamentous actin. For eNOS localization, HUVECs were fixed in −20 °C methanol for 2 min and then processed as previously described^44, 56^. Nuclei were stained with 4′,6-diamidino-2-phenylindole (DAPI). Images were obtained with a Zeiss LSM 510 Meta confocal microscope. The area occupied by lamellipodia was quantified on fixed phalloidin-stained cells by using the NIH Image J program, and expressed as the percent of the total cell area.

### Lentiviral production and transduction

Lentiviral vectors expressing CherryRed or GFP were used. Production of lentiviruses was performed by plasmid transfection into HEK293T cells as previously described^55^. The titer of the lentiviral vector preparations was 1–4 × 10^9^ transducing units/ml. Transduction with GFP or CherryRed was performed at a multiplicity of infection (MOI) of 10. HUVECs were transduced overnight in the presence of 0.5 μg/ml polybrene and re-fed with fresh medium the next day.

### Three-dimensional (3-D) spheroid sprouting assay

HUVECs (1.000 cells/well in low-attachment 96-well round bottom plates) were incubated overnight in the growth medium containing 20% methylcellulose to form spheroids. For mitotic inactivation, mitomycin (500 ng/ml) was added to this medium. Spheroids were then embedded into collagen gels as previously described^57^. Images were acquired with an Olympus U-CMAD3 microscope equipped with an Olympus digital camera 18 h later. In-gel angiogenesis was quantified by measuring the cumulative length of all of the capillary sprouts originating from individual spheroids using the NIH Image J program. At least, 10 randomly selected spheroids *per* experimental group were measured in each experiment. To obtain mosaic spheroids, male and female HUVECs transduced with GFP or Cherry Red were mixed at a 1:1 ratio. Images were acquired 18 h later using a Zeiss LSM 510 Meta confocal microscope.

### Reagents and Antibodies

All tissue culture reagents were from Euroclone SpA except ECGS and heparin (Sigma Aldrich, cat #E2759 and #H3149, respectively). *N* ^G^-Nitro-L-arginine methyl ester (L-NAME, cat #N5751), methylcellulose (cat #M0512), FITC-labelled phalloidin (cat #P5282), and DAPI (cat #D9542) were from Sigma Aldrich; collagenase (cat #17454) and rat tail collagen I (cat #47254) from Serva; recombinant human VEGF_165_ (cat #100) from Peprotech; mitomycin (cat #BIA-M1183) from Tebu-bio. Primary antibodies used were: mouse monoclonals anti-eNOS (BD Transduction Laboratories, cat #610296) and anti-β-actin (Sigma Chemicals, cat #A2228), and rabbit polyclonal anti phospho-eNOS (Ser1177) (Cell Signalling Technology, cat #9571). HRP-conjugated secondary antibodies were from Dako (cat #P0260 and #P0399 for rabbit anti-mouse and swine anti-rabbit antibodies, respectively). A goat anti-mouse CY3 (cat #29-0382-75, GE Healthcare) was used in immunofluorescence experiments to detect eNOS.

### Statistical procedures

Unless otherwise indicated, data are the mean ± s.e.m of at least 3 independent experiments. Statistical significance was determined by unpaired Student’s *t*-test or one-way analysis of variance (ANOVA) followed by Bonferroni’s multiple comparison test using the GraphPad Prism version 5.00 software.

## Electronic supplementary material

Supplementary information
