# Supplementary material for: Sex-specific eNOS activity and function in human endothelial cells
Source: Sci Rep. 2017 Aug 29;7:9612. doi: 10.1038/s41598-017-10139-x (PMC5575132; doi:10.1038/s41598-017-10139-x)
Supplement: Supplementary file 1 — Supplementary information [file 41598_2017_10139_MOESM1_ESM.pdf]

Sex-specific eNOS activity and function in human endothelial cells

Maria Grazia Cattaneo[1,*,#], *Claudia Vanetti*[1,*], *Ilaria Decimo*[2], *Marzia Di Chio*[2], *Giuseppe Martano*[3], *Giulia Garrone*[4], *Francesco Bifari*[5], *Lucia Maria Vicentini*[1,#]

(1) Department of Medical Biotechnology and Translational Medicine,

Università degli Studi di Milano, 20129 Milano, Italy;

(2) Department of Diagnostics and Public Health, Università di Verona, 37134 Verona, Italy;

(3) Institute of Neuroscience, CNR, 20129 Milano, Italy;

(4) Fondazione IRCCS, Istituto Nazionale dei Tumori, 20133 Milano, Italy;

(5) Laboratory of Cell Metabolism and Regenerative Medicine,

Department of Medical Biotechnology and Translational Medicine,

Università degli Studi di Milano, 20129 Milano, Italy

M.G. Cattaneo, C. Vanetti, I. Decimo, M. Di Chio. G. Martano,
G. Garrone, F. Bifari, L. M. Vicentini

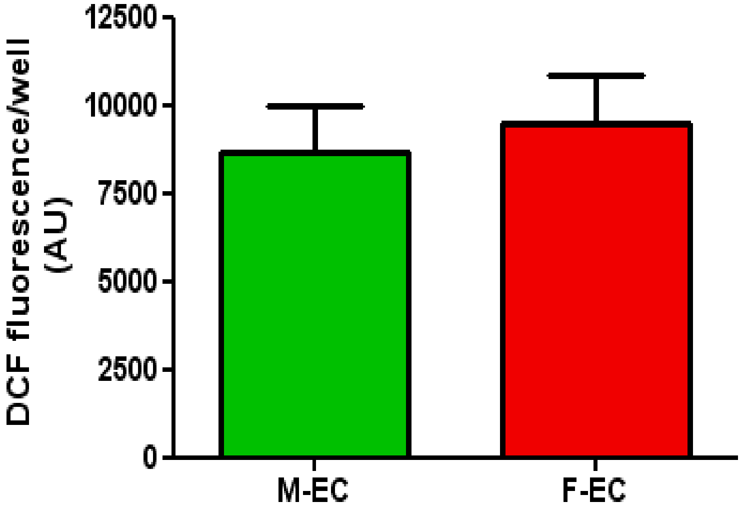

Fig. S1. ROS content was evaluated by measuring cell-associated fluorescence (expressed as arbitrary unit, AU) as described in the Supplementary Methods. p = 0.683, n=13.

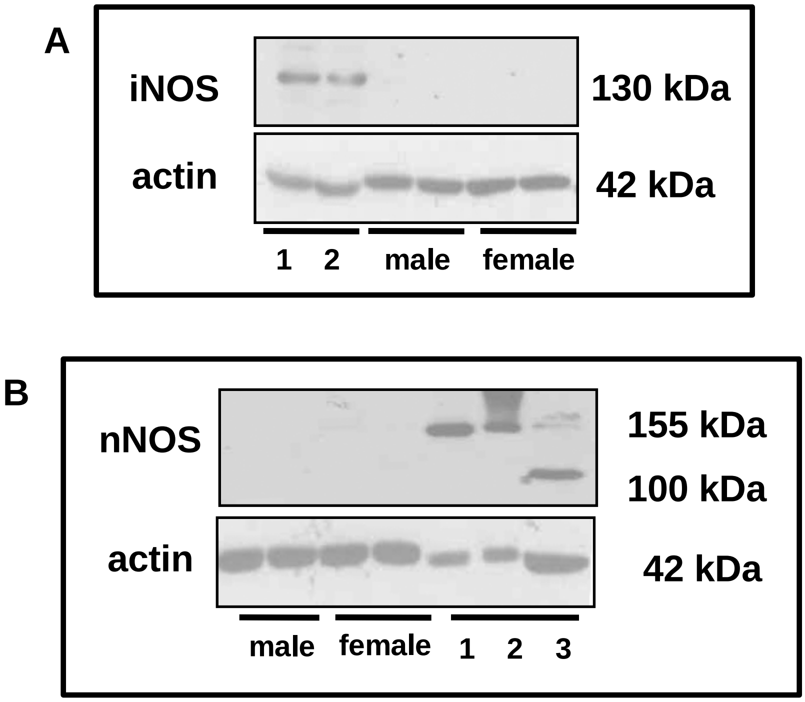

Fig. S2 (**A**) A representative immunoblotting showing total iNOS protein expression in 2 independent male and female EC lysates (20 µg/lane). Lanes 1 and 2: mouse macrophages stimulated with LPS as described in the Supplementary Methods. β-actin was used as a loading control.

Fig. S2 (**B**) A representative immunoblotting showing total nNOS protein expression in 2 independent male and female EC lysates (20 µg/lane). Lane 1: mouse brain cortex (7,5 µg/lane); lane 2: rat hippocampus (7,5 µg/lane); lane 3: human glioblastoma (U87MG cell line, 20 µg/lane). β-actin was used as a loading control.

In mouse and rat homogenates, the expected 155 kDa band was clearly detectable. In human U87MG cells, a weak band was present at 155 kDa whereas a 100 kDa band was evident. The expression of nNOS in U87MG cells has been reported in the literature[1] but the corresponding MW has not been shown. However, a nNOS isoform corresponding to a 100 kDa band has been described in human cells[2]. Nevertheless, neither the 155 nor the 100 kDa bands were detectable in EC lysates.

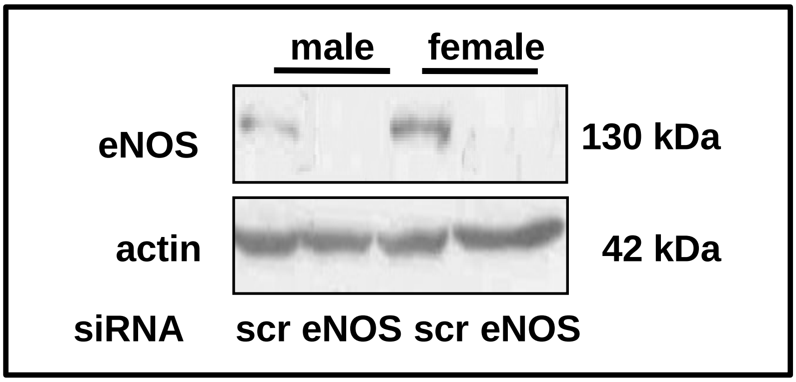

Fig. S3. A representative immunoblotting showing total eNOS protein expression in male and female EC lysates prepared 48h after transfection with scrambled (scr) or eNOS siRNA. β-actin was used as a loading control.

Supplementary Methods

Determination of Reactive Oxygen Species (ROS). Male and female HUVECs were plated at a density of $1.5x10^4$ cells/well in black 96-well microplates, and loaded for 30 min at 37°C in the dark with the fluorescent dye 5(6)-Carboxy-2'7'-dichlorofluorescein diacetate (CM-DCFA, 10 μM) in HBSS buffer (Hepes 25 mM pH 7.4, NaCl 120 mM, KCl 5.4 mM, $CaCl_2$ 1.8 mM, $NaHCO_3$ 25 mM, glucose 15 mM) containing 1% FBS[3]. Cell-associated fluorescence was assessed by means of a multiplate reader with excitation and emission wavelengths of 485 nm and 530 nm, respectively (Victor™, PerkinElmer).

Immunoblotting. Western blots for neuronal and inducible NOSs (nNOS and iNOS, respectively) were carried out on total EC lysates prepared in Laemmli sample buffer containing 1 mM sodium orthovanadate. Equal amounts of proteins (20 μg/lane) were separated by 8% SDS-PAGE, and then transferred onto nitrocellulose membranes following standard procedures. Membranes were blocked for 1 h with 5% milk in Tris-buffered saline containing 0.05% Tween-20 (TBS-T), and probed overnight at 4°C with the following primary antibodies diluted as indicated: mouse monoclonals anti-nNOS (BD Transduction Laboratories, cat #610309, 1:1.000 in 5% milk in TBS-T) and anti-β-actin (Sigma Aldrich, cat #A2228, 1:1.000 in 5% milk in TBS-T), and rabbit monoclonal anti-iNOS (Abcam, cat #178945, 1:1.000 in 5% milk in TBS-T). After incubation with the appropriate HPR-conjugated secondary antibody (1:10.000 in 5% milk in TBS-T), immunoreactive bands were visualized by chemiluminescence (LiteAblot Turbo, EuroClone). Full length blots are shown in Figures 2 and 3. Positive controls are: mouse macrophages stimulated for 24 h with lipopolysaccharide (LPS, 1 μg/ml) (kindly provided by Silvia Franchi, Dept of Pharmacological and Biomolecular Sciences, Università degli Studi di Milano, Italy) for iNOS; a mouse brain cortex homogenate (a kind gift of Irene Corradini, Dept of Medical Biotechnology and Translational Medicine, Università degli Studi di Milano, Italy), a rat hippocampus homogenate (kindly provided by Raffaella Molteni, Dept of Medical Biotechnology and Translational Medicine, Università degli Studi di Milano, Italy), and a human glioblastoma U87MG cell lysate (prepared in our

laboratory) for nNOS. Anti-iNOS and anti-nNOS antibodies were a kind gift of Cristiana Perrotta, Dept of Biomedical and Clinical Sciences "Luigi Sacco", Università degli Studi di Milano, Italy.

References

1 Kashiwagi, S. *et al.* Perivascular nitric oxide gradients normalize tumor vasculature. *Nat Med* **14**, 255-257, doi:10.1038/nm1730 (2008).

2 Papadaki, M., Tilton, R. G., Eskin, S. G. & McIntire, L. V. Nitric oxide production by cultured human aortic smooth muscle cells: stimulation by fluid flow. *Am J Physiol* **274**, H616-626 (1998).

3 Cattaneo, M. G. *et al.* Chronic nitric oxide deprivation induces an adaptive antioxidant status in human endothelial cells. *Cellular Signalling* **25**, 2290-2297, doi:10.1016/j.cellsig.2013.07.026 (2013).
